# Surgery

**Published:** 1900-03

**Authors:** 


**Surgery.** By Thomas Pickering Pick. Pp. xix., 1176. London: Longmans, Green, and Co. 1899.

Mr. Pick tells us that this book is the substance of the lectures on surgery delivered by him for fifteen years at St. George's Hospital, modified, of course, as surgery has advanced, and mainly intended for the use of students. He explains that he is only able, in the space at his command, to give his own views as to the nature of the injuries and diseases of which the book treats, and is not able to refer to the views of others; and that he cannot do more than recommend the methods of treatment he has himself found most useful, with merely an incidental allusion to other methods. But on perusal of the book we are glad to find that it very well represents the practice of surgery of the present day, for we think that this is of much more use to students than Mr. Pick's own views; though, no doubt, to old St. George's students the views of their teacher of surgery would be of particular interest. But what the student requires, in a text-book of surgery, is a representation of the generally-received views as to the nature of injuries and diseases and their treatment. Students not only have to prepare for the future treatment of patients, but they have to encounter a body of gentlemen at the College of Surgeons who may not hold Mr. Pick's particular theory, or adopt his line of treatment. We certainly think it is very important for every writer of a surgical text-book, and every lecturer of surgery in a Medical School, to teach his own views however novel they may be; but we also hold very strongly that he should make quite clear what the generally-received view is, and wherein his own differs from it.

Mr. Pick's book is a very excellent one—well written, clearly arranged, and with accounts of quite modern views, and methods of treatment. It is one we can strongly commend to students. We may wish that here and there a little more space might have been given to the elaboration of some diseases, which are considered in very bare outline; but, unfortunately, this is the inevitable result of having to include so large a subject in so small a space.

We notice that surgical diseases of the female generative organs receive more attention than usual in a work of this kind, and the information given is valuable. We are glad to see that reference is made to Dean's method of exploration for cerebral abscess. Calot's treatment of deformity from Pott's disease of the spine is also mentioned, but Mr. Pick wisely declines to express any opinion as to its merits at the present time. The chapters on fractures and dislocations should be of special value, as Mr. Pick has written one of the best books on the subject. We notice reproductions of a few skiagrams illustrating this subject. We think the drawings of pathological specimens and morbid clinical conditions are not very good; and we strongly object to such an indefinite way of writing of a method of treatment as the following directions for operating for anal fistula. Mr. Pick says: "If the walls of the main sinus are much thickened, as is usually the case, they should be dissected away with scissors or a knife, and a clean raw surface left. If this is thoroughly done, the surfaces of the wound may now be brought together by sutures, and the convalescence materially hastened; but in most cases it is better to dust the wound with iodoform, and then pack it with gauze soaked in iodoform emulsion." If suturing hastens convalescence, why is it better in most cases not to employ it? Let the method be either advised or condemned for some definite reason. We cannot, of course, expect the relative merits of various methods of treatment to be discussed at length in this work. The student has not the difficult task of deciding which method of treatment to adopt out of several possible methods, and hence the information of such a text-book as this is sufficient for them, if only the advice given is clear and consistent.

Taken as a whole, the book is one which we can safely place in the hands of students, and which we feel sure will become very popular with them.

